# Measuring the mutual diffusion coefficient of heavy water in normal water using a double liquid-core cylindrical lens

**DOI:** 10.1038/s41598-018-30650-z

**Published:** 2018-08-22

**Authors:** Weidong Meng, Yan Xia, Yan Chen, Xiaoyun Pu

**Affiliations:** 1 Department of Physics, Yunnan University, Kunming, Yunnan 650091 China; 2 International Research Center for Optoelectronic Energy Materials Yunnan University, Kunming, 650091 China

## Abstract

The mutual diffusion coefficient of heavy water in normal water is measured over a temperature range of 20 to 40 °C using a novel method called the shift of equivalent refractive index slice (SERIS). The measured values range from 1.9086 × 10^−5^ to 3.0860 × 10^−5^ cm^2^/s and fit the Arrhenius equation well, and the calculated data from the equation are consistent with the literature values obtained by the interference method. The SERIS method is based on a double liquid-core cylindrical lens (DLCL); the front liquid core of the DLCL is used as both a liquid diffusion cell and a key imaging lens, and the resolvable minimum of liquid refractive index is *δn* = 6.15 × 10^−5^. The rear liquid core is used as an aplanatic lens, and the transversal spherical aberration is less than 1 μm. The SERIS method provides a new way to measure mutual diffusion coefficients of liquid and has the following advantages: visual measurement, use of a simplified device, and easy operation.

## Introduction

Heavy water (deuterium oxide, D_2_O), which is used as a moderator and coolant in nuclear reactors, plays an important role in the production of nuclear energy^1^; as an isotope tracer material, heavy water is used to detect changes in chemical and physiological activities^2^. In general, heavy water is extracted from normal water (a mixed solution of H_2_O, DHO and D_2_O)^3^ and the study of diffusion processes and the measurement of the mutual diffusion coefficient (*D* value) of heavy water in normal water are important, particularly for heavy water separation and its applications^4–6^. The traditional methods for measuring the *D* value of heavy water in normal water include interferometry^7^, a diaphragm cell^8^, Taylor dispersion^9^ and nuclear magnetic resonance (NMR)^5^. The common disadvantages of these methods include that (1) long times (>2 h) are required to obtain experimental data and that (2) the diffusion process cannot be observed directly. In addition, the interferometry method requires extremely strict experimental environments^10^; a diaphragm cell needs to be calibrated^8^; the Taylor dispersion method uses a spiral capillary with a length of up to several metres, and the mobile phase is required to flow through the round cross section at a constant flow rate, which will bring some inconvenience to measure the diffusion coefficient^11^. NMR can only measure certain substances^5^.

To overcome these disadvantages, we have designed and fabricated a non-symmetrical liquid-core cylindrical lens^12,13^ that can be used to measure the liquid diffusion coefficient quickly and accurately and can observe dynamic diffusion processes directly^12,13^. Furthermore, a double liquid-core cylindrical lens (DLCL) has been recently designed and fabricated^14^, with a higher refractive index (RI) sensitivity, better RI resolving power and smaller spherical aberration (SA) than that of a corresponding liquid-core cylindrical lens. Using the DLCL, the *D* values of heavy water in normal water at a temperature range from 20 to 40 °C have been measured by a novel method called the shift of equivalent RI slice (SERIS), and the measured *D* values are fitted by the Arrhenius equation^15^. The calculated *D* values from the fitted equation are then compared with literature values using an interferometry method^7^. The method, results and discussion are reported in this paper. Using the DLCL and a CMOS camera, the entire diffusion process is visualized and displayed in the attached Visualization file.

## Principle and Methods

### Imaging principle for a DLCL filled with different solutions

**Figure 1:**
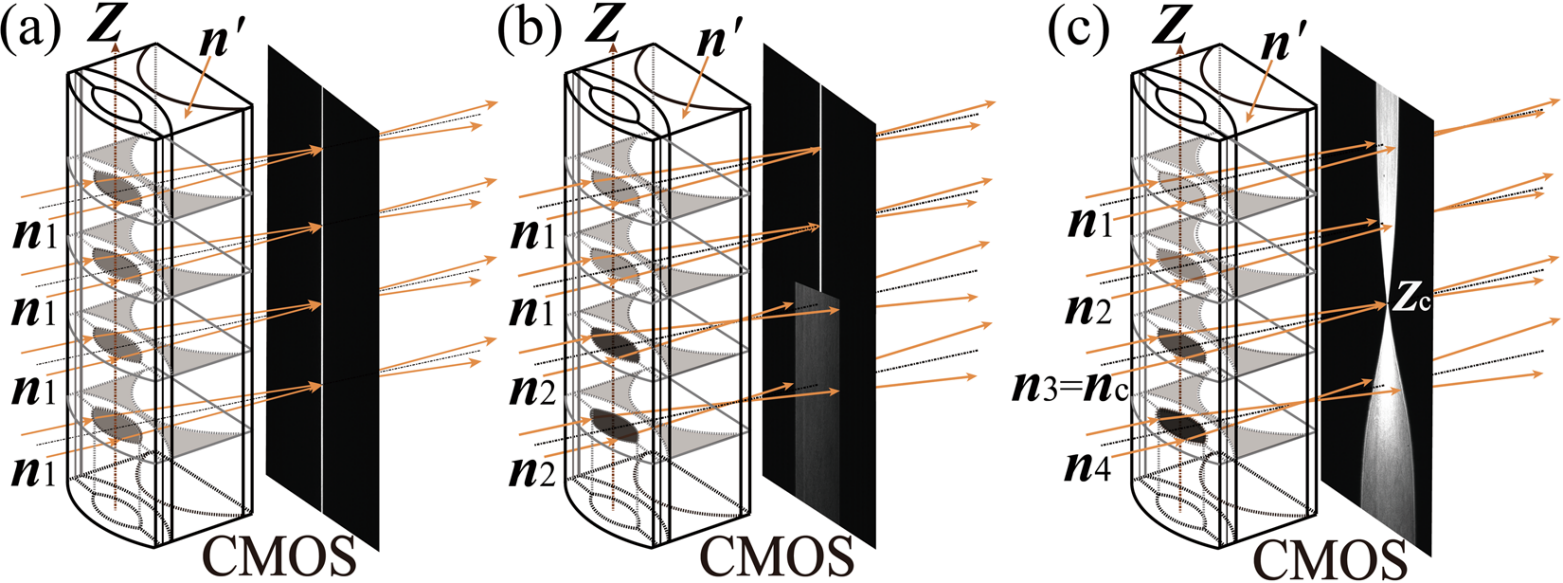
Diagram of the designed DLCL, and illustration of the imaging principle for a DLCL filled with different liquids in the front liquid core. (**a**) Filled with a uniform liquid with RI = *n*_1_. (**b**) Filled with two different liquids, *n*_1_ < *n*_2_. (**c**) An RI gradient distribution of the filled liquid forms along the *Z*-axis, *n*_1_ < *n*_2_ < *n*_3_ = *n*_c_  < *n*_4_.

The DLCL is composed of two liquid-core cylindrical lenses, as shown in Fig. 1. The front lens of the DLCL is used as both the diffusion cell and as a key imaging element, and the RI = *n*_i_ of the liquid filled in its core can be measured in the way of spatial resolution; the rear lens of the DLCL is used as an aplanatic component, and either the RI position of SA or the SA over an RI range caused by the front lens can be regulated by filling the rear liquid core with a liquid for which RI = *n*′ is known.

When monochromatic collimated light beams pass through the DLCL, if the front liquid core of the DLCL is filled with one uniform liquid, the focal lengths at different heights (*Z*_i_) of the DLCL are the same. A CMOS chip positioned at the focal plane will receive a sharp image of a line, as shown in the right side of Fig. 1(a). If two liquids with different RIs (e.g., *n*_1_ and *n*_2_) are loaded into the front liquid core of the DLCL successively, when collimated light beams from the upper part are imaged clearly on the CMOS chip, the beams from the lower part are imaged diffusely on the chip, as shown in the right side of Fig. 1(b). Once the two liquids contact each other, the diffusion process commences, and the time of contact of the two solutions is defined as the onset of diffusion (*t* = 0). A dynamic concentration gradient distribution for the two solutions is formed gradually along the *Z*-axis (diffusion direction), corresponding to a measurable RI gradient distribution. The collimated light beams passing through the DLCL project a dynamic “beam waist” image on the CMOS as shown in the right side of Fig. 1(c), and a sharp point (*Z*_c_) on the CMOS defines a liquid slice so that RI = *n*_3_ = *n*_c_. The dynamic images reflect the diffusion process, based on Fick’s second law^16^, the *D* value can be determined by calculating the shift rate of the selected liquid slice in the front liquid-core diffusion lens.

### Theoretical calculation of the diffusion coefficient

The diffusion of two solutions along the liquid core of the cylindrical lens (*Z*-axis) is considered a one-dimensional free diffusion process that follows Fick’s second law. If *C*(*Z*, *t*) is the solution concentration at diffusion time *t* and position *Z*, the equation can be written as

1 $$\frac{\partial C(Z,\,t)}{\partial t}=\frac{\partial }{\partial Z}(D\frac{\partial C(Z,\,t)}{\partial Z}).$$

Let the initial concentrations be *C*_1_ and *C*_2_ on two sides of the interface (*Z* = 0) before the beginning of diffusion. In the case of a dilute solution, the *D* value in Eq. 1 is a constant, and its solution is an error function that can be expressed as^9,16^

2 $$C(Z,\,t)=f[n(Z,\,t)]=\frac{{C}_{1}+{C}_{2}}{2}+\frac{{C}_{1}-{C}_{2}}{2}erf(\frac{Z}{2\sqrt{Dt}}),$$

where $erf(Z/2\sqrt{Dt})$ is the Gauss error function and *n*(*Z*, *t*) is the spatial and temporal distribution of the RI. The relationship between *C*(*Z, t*) and *n*(*Z*, *t*) can be obtained in advance by the experimental method. In the actual measurements, due to intermolecular attractions, the contact interface of two solutions cannot be an exact horizontal plane (*Z* = 0), which leads to difficulty in precisely positioning the contact interface, and a value relative to the contact interface is represented as the deviation, Δ*Z*^13^. If the position of the selected liquid slice is *Z*_i_, the collimated light passing through the slice can be sharply imaged on a CMOS chip, and *Z*_i_ can be modified to *Z*_i_ + Δ*Z*. Eq. 2 can be revised as

3 $${Z}_{i}=2\sqrt{Dt}\cdot erfinv\{(f[{n}_{c}({Z}_{i},\,t)]-\frac{{C}_{1}+{C}_{2}}{2})/(\frac{{C}_{1}-{C}_{2}}{2})\}-{\rm{\Delta }}Z.$$

Let ${\rm{\Omega }}=(f[{n}_{c}({Z}_{i},\,t)]-({C}_{1}+{C}_{2})/2)/(({C}_{1}-{C}_{2})/2)$, *erfinv* (Ω) is the inverse of the error function, which is a constant for the selected liquid slice. Since diffusion is a dynamic process, the position of the liquid slice *Z*_i_ will drift slowly with diffusion time, and a slope *k*_1_ is obtained by linearly fitting the experimental data for *Z*_i_ and *t*^1/2^. The diffusion coefficient *D* can be obtained by $D={k}_{1}^{2}/4{{\rm{\Omega }}}^{2}$. We call the measurement method introduced above the SERIS.

## Results

The RIs of heavy and normal water at room temperature (25 °C) are 1.3282 and 1.3330, respectively, and the difference is only 0.0048 To measure the *D* value with the SERIS method, it is necessary for the DLCL to have a high RI resolving power, and the resolvable minimum of RI variation (*δn*) should be much smaller than 0.0048. *δn* is determined by^14^

4 $$\delta n={\frac{{\rm{\Delta }}n}{({\rm{\Delta }}f/\delta f)}|}_{{\rm{\Delta }}n=0.0002},$$

where Δ*f* is the RI sensitivity, defined as the change in focal length caused by the change in RI (Δ*n*); the measurement error of the focal length (*δf*) depends mainly on the geometric depth of field (DOF)^14^. For the DLCL used, Δ*f* ≥ 306.9 μm in the range of *n* = 1.3282 to 1.3330 when *n*′ (liquid filled in the rear liquid core) is fixed at 1.3990 (the reason is explained in the discussion), δ*f* < 99.3 μm, and δ*n* is thus smaller than 6.15 × 10^−5^ and is satisfied fully in terms of the experimental requirement.

To measure the *D* value as it varies with temperature, the experimental relationship between the concentration and RI of the heavy water aqueous solution at different temperatures, *C*(*T*) = *f* [*n*(*T*)] should be known in advance. Thus, all experiments were performed in an air-conditioned room with a temperature range of 20 to 40 °C. Eleven groups of mixed solutions of heavy and normal water were prepared for each experimental temperature, and the concentrations of the mass fraction (*C*) were prepared using an electronic auto-balance with 0.0001 g precision. The related RI values (*n*) were measured using an Abbe Refractometer (Shanghai optical instrument factory, Shanghai, China) with a precision of 0.0002 at different temperatures. The fitting results showed a good linear relationship and are listed in the third column of Table 1.

**Table 1:** Experimental relationship between the concentration and RI of mixed solutions at different temperatures.

Temp./°C	Temp./K	Fitting expression/%	*R* ^2^
20	293	*C* = −212.5663*n* + 283.3988	0.9991
25	298	*C* = −206.2113*n* + 274.8735	0.9992
30	303	*C* = −216.7846*n* + 288.8353	0.9992
35	308	*C* = −229.0474*n* + 305.0226	0.9995
40	313	*C* = −219.8241*n* + 292.6323	0.9992

**Figure 2:**
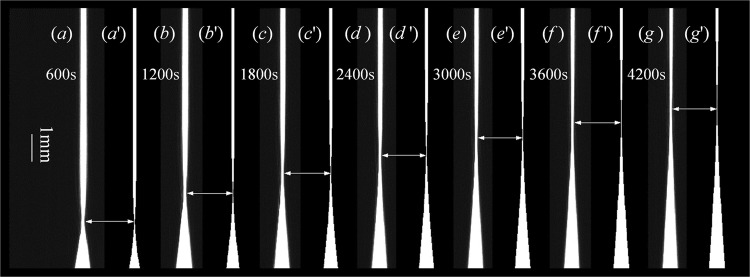
Diffusion images recorded using a CMOS camera at different diffusion times and simulated images of the corresponding times. The experimental temperature is 25 °C, and focal positions (*Z*_i_) are marked by arrows. The narrowest image width corresponds to the liquid layer of RI = *n*_c_ = 1.3325. (***a***–***f***) are experimental diffusion images, and (***a*’**–***f*’**) are the corresponding simulated diffusion images from tracing beams passing through the DLCL.

Laser light (λ = 589.0 nm) was beam expanded and collimated by a pair of lenses. After passing through the DLCL, the light was projected onto a CMOS chip (4096 × 3072 pixels^2^, 5.5 × 5.5 μm^2^ for each pixel) mounted on a 2D translation stage. Pure normal water (*n* = 1.3330) was used in the front core of the DLCL to calibrate the measurement system. After calibrating, the CMOS chip was fixed at a specific position where the beams passing through a selected liquid slice of *n*_c_ = 1.3325 focused sharply on the chip (the selection rule for the slice is explained in the next part). The heavy water (purity 99.8%, produced by ARMAR Co., LTD) was injected into the front core of the lower half of the DLCL and left for 5–10 minutes to dampen liquid turbulence. Then, normal water (deionized and distilled pure water) was slowly introduced into the upper half of the DLCL along the inner wall to ensure no obvious turbulence current in the two liquids. The air-conditioned room was adjusted to 25 °C, and diffusion images were obtained starting at *t* = 600 s with an interval of 120 s. Some of the images obtained from 600 to 4200 s are shown in Fig. 2(a–g); the focal position (*Z*_i_) varied with diffusion time (*t*) is listed in Table 2.

**Table 2:** Data for the focal position varied with diffusion time.

*t*/s	*Z*_i_/pixel	*Z*_rdm_/pixel	*t*/s	*Z*_i_/pixel	*Z*_rdm_/pixel	*t*/s	*Z*_i_/pixel	*Z*_rdm_/pixel
600	397	394	1920	676	705	3240	894	873
720	435	407	2040	697	677	3360	919	949
840	461	439	2160	719	708	3480	936	941
960	493	518	2280	739	736	3600	960	955
1080	519	516	2400	760	763	3720	975	987
1200	554	541	2520	775	790	3840	990	954
1320	583	614	2640	794	756	3960	1005	1027
1440	609	589	2760	816	846	4080	1016	1009
1560	629	614	2880	834	867	4200	1026	1065
1680	638	661	3000	849	859			
1800	658	619	3120	872	904			

A random integer between −39 and 39 is added to the focal position (*Z*_rdm_) to estimate the experimental deviation.

The focal position *Z*_i_ is linearly fitted with *t*^1/2^ by the least square method, and the result is expressed as

5 $${Z}_{i}=86.451\sqrt{{t}_{i}}-7.587\,(\mu m).$$

The linear correlation coefficient used for fitting in Eq. 5 is 0.9964. Let *C*_1_ = 0, *C*_2_ = 1, and *C* = *f*(*n*_c_ = 1.3325) = 0.0969 in Eq. 2, so that Eq. 3 can be written as

6 $${Z}_{i}=2\sqrt{D{t}_{i}}erfinv({\rm{0.8062}})-\,{\rm{\Delta }}Z.$$

*D* = 2.2133 × 10^−5^ cm^2^/s at *T* = 25 °C, which is determined by comparing Eq. 5 with Eq. 6. If *D* = 2.2133 × 10^−5^ cm^2^/s and *C*(*Z, t*) satisfies Eq. 2, diffusion images that varied with time were simulated by tracing beams passing through the DLCL, which is shown in Fig. 2(a’–g’). When the simulations are compared with experimental images, it is clear that the draft rate of the “waist” of the diffusion image is the same in the two groups of images, which demonstrates that the SERIS method is useful and the acquired diffusion data are reliable.

**Figure 3:**
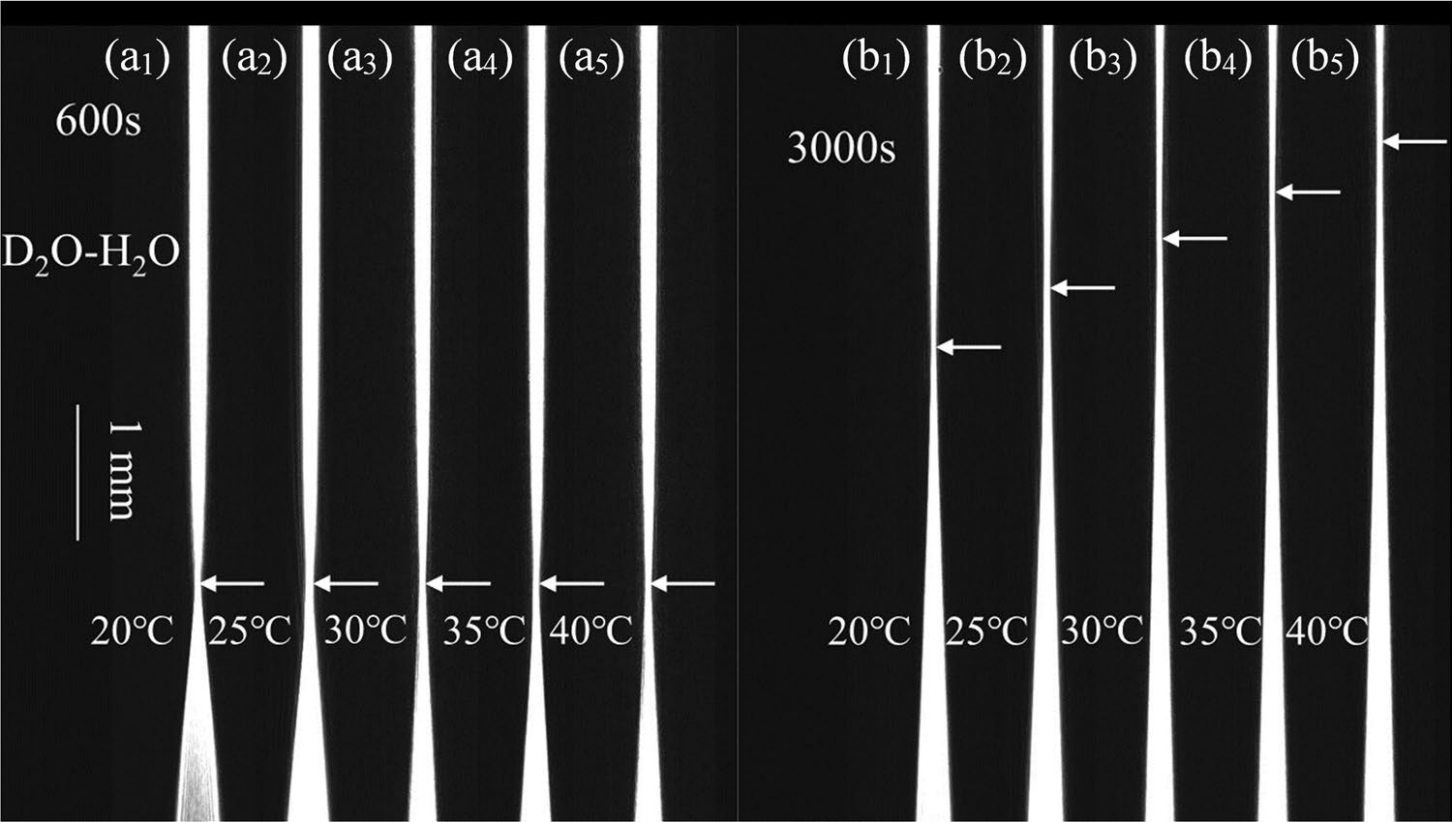
Diffusion images at various temperatures and different times. The dynamic diffusion process is shown in the Visualization file.

Liquid diffusion is very sensitive to temperature, and the dependence of the *D* value on temperature was measured using the method introduced above. The results are listed in Table 3. It is clear from Table 3 that the measured diffusion coefficients increase monotonously with experiment temperature. The positions of the equivalent RI slices in the CMOS chip for different temperatures were adjusted so that their focal points at diffusion time *t* = 600 s were located at the same position shown in Fig. 3(a). Diffusion images at the same diffusion time (*t* = 3000 s) are shown in Fig. 3(b), which indicates that the shift rate of the selected RI slice at high temperatures is faster than that at low temperatures. The entire dynamic diffusion process is shown in the attached Visualization file, which enables vivid visualization of the microscopic diffusion process.

**Table 3:** Measured D values of heavy water diffusing in normal water at various temperatures.

*T*/°C	RI (*n*_c_)	Mass fraction (%)	Fitting expression	*D* × 10^−5^ (cm^2^/s)
20	1.3328	9.04	*Z* = 82.684*t*^1/2^−59.967	1.9086
25	1.3325	9.69	*Z* = 86.451*t*^1/2^−7.587	2.2133
30	1.3319	9.99	*Z* = 91.713*t*^1/2^ + 45.338	2.5584
35	1.3313	9.18	*Z* = 99.572*t*^1/2^−27.743	2.8034
40	1.3308	9.04	*Z* = 103.060*t*^1/2^ + 57.916	3.0860

The measured *D* values were fit with the Arrhenius equation^15^, a well-known formula describing the dependence of the *D* value on temperature (*T*), which can be expressed as

7 $$\mathrm{ln}\,(D)=\frac{-{E}_{a}}{{k}_{B}T}+A.$$

**Figure 4:**
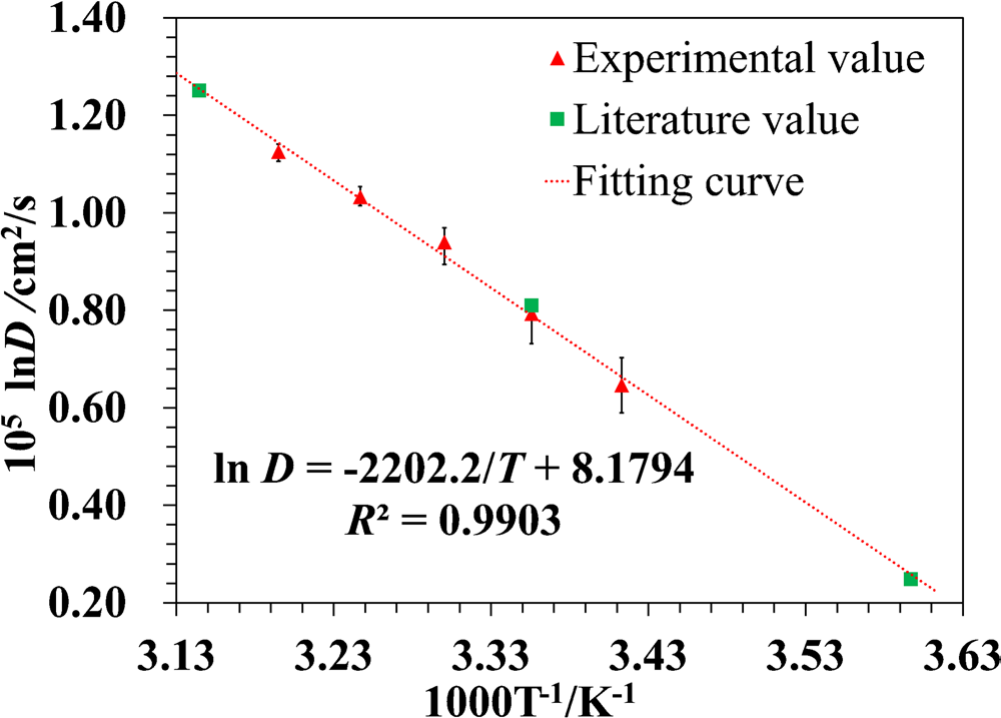
Fitted *D* values varied with temperature. Triangles represent experimental data; the squares represent the literature values; the dotted line indicates the linear fit of the experimental data. The error bars were determined by 5 independent measurements.

Where *k*_*B*_ is the Boltzmann constant, *E*_*a*_ is the activation energy, and *A* is the pre-exponential factor. The linear fitting result with *R*^2^ = 0.9905 is shown in Fig. 4, which yields *E*_*a*_ = 3.0406*10^−20^ J and *A* = 8.1794. By substituting the values of *E*_*a*_, *A*, and *T* = 5, 25, and 45 °C into Eq. 6, the calculated *D* values are *D*_(T=5°C)_ = 1.294 × 10^−5^, *D*_(T=25°C)_ = 2.202 × 10^−5^ and *D*_(T=45°C)_ = 3.505 × 10^−5^ cm^2^/s, respectively. For comparison, the *D* values in the literature^7^ measured using interferometry are *D*_*lit*(T=5°C)_ = 1.281 × 10^−5^, *D*_*lit*(T=25°C)_ = 2.248 × 10^−5^ and *D*_*lit*(T=45°C)_ = 3.491 × 10^−5^ cm^2^/s, which are consistent with our values.

## Discussion

**Figure 5:**
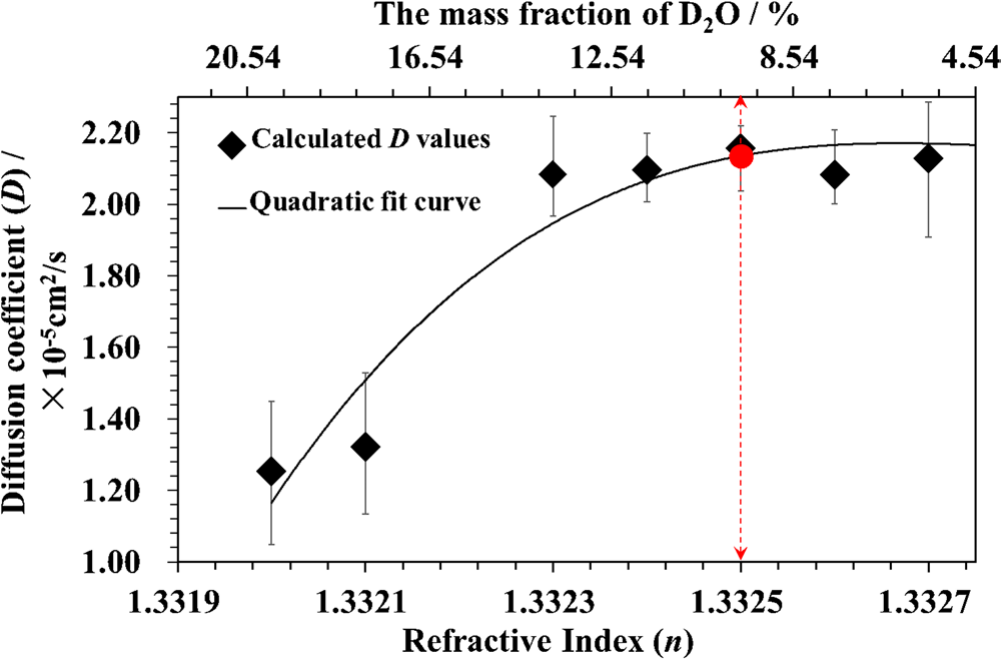
The calculated *D* values at T = 25 °C varied with different liquid slices. The dotted arrow indicates the liquid slice of *n*_c_ = 1.3325 and related mass fraction. The error bars were determined by 5 independent measurements.

Two questions remain to be answered. The first question is how should the liquid that fills the rear liquid core of the DLCL be chosen? The rear liquid core is used to eliminate the SA caused by the front liquid core, which is the main aberration in the measurement. The RI of the diffusion solution varied from *n* = 1.3282 to 1.3330 for the experiment with heavy water diffusing in normal water; the solution for which the RI is *n*′ = 1.3990 is chosen by aberration analysis^14^, so that the SA of the DLCL is close to zero at *n* = *n*_c_ = 1.3295 and is less than 1 µm over the range of *n* = 1.3282 to 1.3330. The second question is what is the rule governing the selection of the liquid slice? To answer this question, liquid slices with different RIs were selected, and similar experiments were performed at 25 °C for a liquid slice of *n*_c_ = 1.3325. The experimental results are shown in Fig. 5, which indicates that the calculated *D* values depended on the solution concentrations when the RI of the selected liquid slice was smaller than 1.3324, corresponding to the concentration *C*′ = 11.76%. When the selected RI is near 1.3330, corresponding to the initial concentration *C*_1_ = 0 (pure normal water), the *D* values tend to be a stable constant. Since Eq. 2 is the calculation basis of the *D* value, which is the solution for Eq. 1 under the precondition that the *D* value is independent of the solution concentration, it is unfeasible for the *D* value to depend on the solution concentration. Therefore, when the SERIS method is applied to measure the *D* value for binary solutions, the rule in selecting the liquid slice is that its concentration should be close to the initial value (*C*_1_ or *C*_2_ in Eq. 2).

For the SERIS method, the experimental deviation is mainly caused by error in reading the focal position (*Z*_i_), which may occupy several pixels along the diffusion direction (*Z*-axis) but has the same image width. This phenomenon becomes obvious when the concentration gradient of the diffusion solution is small, as shown in Fig. 2(e–g). The deviation of the focal position caused by the concentration can be written as

8 $$\delta Z=\frac{2\sqrt{\pi Dt}}{{C}_{1}-{C}_{2}}exp{(\frac{Z}{2\sqrt{Dt}})}^{2}\delta C,$$

which is deduced from Eq. 2. Using the data obtained at *T* = 25 °C as an example, let *C*_1_ = 0, *C*_2_ = 1, *D* = 2.2133 × 10^−5^ cm^2^/s, *t* = 2400 s, *Z* = 702 μm, and *δC* = 1.27%, which is calculated by assuming a resolvable RI minimum of *δn* = 6.15 × 10^−5^. The calculated δ*Z* is 420 μm, corresponding to ∼77 pixels along the *Z*-axis. A random integer between −39 and 39 is then added to the measured *Z*_i_, which is labelled as *Z*_rdm_ in the 3^rd^, 6^th^ and 9^th^ columns in Table 2. The fitting result between *Z*_rdm_ and *t*^1/2^ is ${Z}_{{\rm{rdm}}}=89.134\sqrt{t}-110.351$ (μm, *R*^2^ = 0.9838), which yields *D* = 2.3528 × 10^−5^ (cm^2^/s). Therefore, the relative deviation caused by the error in reading the focal position is approximately 6.3%.

In summary, using a DLCL, a new measurement method called SERIS has been designed and tested. The diffusion coefficients of heavy water in normal water have been measured over a temperature range of 20 to 40 °C, and the measured values have been well fitted by the Arrhenius equation. The calculated data from the equation are consistent with those obtained by the interference method. The rules governing the selection of both the liquid slice in the front liquid core and the solution in the rear liquid core of the DLCL have been discussed, and the main experimental error has been estimated in the last part of this paper. The SERIS method using a DLCL to measure liquid *D* values has the advantages of visual measurement and rapid testing, and the required RI difference of the two diffusion liquids is small.

## Electronic supplementary material

Description of video

Visualization Video
