# Supplementary material for: Measuring the mutual diffusion coefficient of heavy water in normal water using a double liquid-core cylindrical lens
Source: Sci Rep. 2018 Aug 22;8:12610. doi: 10.1038/s41598-018-30650-z (PMC6105602; doi:10.1038/s41598-018-30650-z)
Supplement: Supplementary file 2 — Visualization Video [file 41598_2018_30650_MOESM2_ESM.doc]

**Supplementary Information**

**Manuscript title：** Measuring the mutual diffusion coefficient of heavy water in normal water using a double liquid-core cylindrical lens

**Author list：** Weidong Meng, Yan Xia, Yan Chen, Xiaoyun Pu.

**Video legends:**

Visualization Video. The effect of temperature on diffusion process. The video shows heavy water diffusing in normal water (*C*_1_=1, *C*_2_=0) from 20°C to 40°C. Those five dynamic diffusion process showed in one video indicate that higher temperature causes more vigorous random molecular motions, leading to a higher diffusion rate. Video refers to the last paragraph from top of page 1 of the article file.
